# Molecular Signatures of Natural Selection for Polymorphic Genes of the Human Dopaminergic and Serotonergic Systems: A Review

**DOI:** 10.3389/fpsyg.2016.00857

**Published:** 2016-06-08

**Authors:** Daniel R. Taub, Joshua Page

**Affiliations:** ^1^Department of Biology, Southwestern University, GeorgetownTX, USA; ^2^School of Medicine, Washington University, St LouisMO, USA

**Keywords:** balancing selection, dopamine, genome scan, genomics, natural selection, population genetics, positive selection, serotonin

## Abstract

A large body of research has examined the behavioral and mental health consequences of polymorphisms in genes of the dopaminergic and serotonergic systems. Along with this, there has been considerable interest in the possibility that these polymorphisms have developed and/or been maintained due to the action of natural selection. Episodes of natural selection on a gene are expected to leave molecular “footprints” in the DNA sequences of the gene and adjacent genomic regions. Here we review the research literature investigating molecular signals of selection for genes of the dopaminergic and serotonergic systems. The gene *SLC6A4*, which codes for a serotonin transport protein, was the one gene for which there was consistent support from multiple studies for a selective episode. Positive selection on *SLC6A4* appears to have been initiated ∼ 20–25,000 years ago in east Asia and possibly in Europe. There are scattered reports of molecular signals of selection for other neurotransmitter genes, but these have generally failed at replication across studies. In spite of speculation in the literature about selection on these genes, current evidence from population genomic analyses supports selectively neutral processes, such as genetic drift and population dynamics, as the principal drivers of recent evolution in dopaminergic and serotonergic genes other than *SLC6A4*.

## Introduction

Several human genes coding for proteins involved in synthesis, reception, transport, or degradation of dopamine and/or serotonin contain well-studied polymorphic nucleotides single-nucleotide polymorphisms (SNPs) or repeat sequences (**Table 1**; for convenience we will refer to these as “neurotransmitter genes” although their gene products are of course not the neurotransmitters themselves). There is a large literature evaluating the possible influence of these genetic variants on diverse aspects of human behavior, personality, and susceptibility to psychiatric disorders (a few recent reviews: Ebstein et al., 2010; Bakermans-Kranenburg and van IJzendoorn, 2011; Balestri et al., 2014; Montag and Reuter, 2014; Gatt et al., 2015; Mileva-Seitz et al., 2015)

**Table 1:** Genes of the human dopaminergic and serotonergic systems putatively under natural selection.

Gene abbreviation	Gene Product	Protein function	Prominent polymorphisms	MAF
*HTR2A*	5-hydroxytryptamine receptor 2A	Serotonin receptor	rs6311: Promoter region SNP	0.44


			rs6313: Exon I synonymous SNP	0.44
			rs36213156: Upstream SNP	0.11
*SLC6A4*	Solute Carrier Family 6 (Neurotransmitter Transporter), Member 4	Serotonin Transporter	5-HTTLPR: Promoter VNTR	0.31


			Intron 2 VNTR	0.26
			rs25531: Upstream SNP	0.14
			rs25532: Upstream SNP	0.08
*TPH1*	Tryptophan hydroxylase 1	Synthesis of serotonin	rs211105: Intron 2 SNP	0.15
			rs1800532: Intron 6 SNP	0.32
			rs7933505: Intron 7 SNP	0.29
*SLC6A3*	Solute Carrier Family 6 (Neurotransmitter Transporter), Member 4	Dopamine transporter	3′ UTR VNTR	0.17
*DRD2*	Dopamine receptor D_2_	Dopamine receptor	rs1800497: Missense SNP in *ANKK1* exon 8^∗^	0.33
			rs1801028: Exon 6 Missense SNP	0.03
			rs 6277: Exon 6 synonymous SNP	0.24
			rs12574471: Intron 1 SNP	0.11
*DRD3*	Dopamine receptor D_3_	Dopamine receptor	rs6280: Exon 3 Missense SNP	0.49
*DRD4*	Dopamine receptor D_4_	Dopamine receptor	Exon 3 VNTR	0.22
*COMT*	Catechol-O-methyltransferase	Degradation of catecholamines, including dopamine	rs4680: Exon 4 missense SNP	0.37
*MAOA*	Monoamine oxidase A	Monoamine neurotransmitter degradation	*MAOA*-LPR: Promoter region VNTR	0.36
*MAOB*	Monoamine oxidase B	Monoamine neurotransmitter degradation	rs5905512: Intron 1 SNP	0.44


Genes of the dopaminergic and serotonergic systems are listed if hypotheses have been advanced that polymorphisms in these genes result from natural selection. MAF, Minor Allele Frequency, the approximate global frequency of the second most common variant. Frequency data for SNPs from dbSNP build 144 (http://www.ncbi.nlm.nih.gov/SNP/, accessed October 2015), for *MAOA* VNTR from Sabol et al. (1998), for 5-HTTLPR from Nakamura et al. (2000) and for other VNTR from ALFRED (http://alfred.med.yale.edu, accessed October 2015). *The rs1800497 SNP has often been discussed in the literature as related to *DRD2*, although subsequent research has revealed it to be within an adjacent gene, *ANKK1*.

The question of why there exists variation for genes affecting human neural function and behavior has engaged many authors (e.g., Keller and Miller, 2006; Nettle, 2006; Crespi et al., 2007; Penke et al., 2007; Verweij et al., 2012). This genetic variation must result from some combination of the basic evolutionary processes of genetic drift, mutation, and natural selection, along with demographic history (including gene flow). Particularly intriguing is the possibility that natural selection has played a role in the development of the genetic variation in neurotransmitter genes, and a variety of selective explanations for these polymorphisms have been proposed (**Table 2**).

**Table 2:** Selective explanations proposed for polymorphisms in human neurotransmitter genes.

Hypothesis	Explanation	Selected references
**Balancing selection**		
Overdominance (aka heterozygote advantage)	Overdominance refers to a situation in which heterozygotes have a greater fitness than either homozygote, therefore maintaining both alleles in the population	Gosso et al., 2008; Crespi, 2009; Costas et al., 2011
Negative frequency dependent selection	In negative frequency dependent selection, phenotypes (and associated genotypes) are favored by virtue of being rare. Rare alleles therefore tend to increase in frequency, maintaining genetic variation	Ding et al., 2002
Environmental heterogeneity balancing selection	Selection favors different phenotypes/genotypes in different circumstances, thereby preserving more than one genetic variant	Ding et al., 2002; Grady et al., 2003; Wang et al., 2004; Crespi et al., 2007; Crespi, 2009; Garcia et al., 2010; Costas et al., 2011
Differential susceptibility (basic)	Different environments favor individuals whose behavioral development is more or less responsive to their social environment	Homberg and Lesch, 2011; Sulik et al., 2015
Differential susceptibility (w bet-hedging)	As with basic differential susceptibility, but producing children with different levels of environmental susceptibility is viewed as a parental bet-hedging strategy	Ellis et al., 2011; Belsky et al., 2015
**Positive selection**		
Ancestral Susceptibility	Major changes in human behavior, diet, etc. have led to changes in selective regimes. Ancestral alleles that were formerly favored are associated in the new selective regimes with dysfunction, and are now being selected against	Ding et al., 2002; Arcos-Burgos and Acosta, 2007; Guo et al., 2007; Carrera et al., 2009; Crespi, 2009


*The authors cited proposed the hypothesis in question for a specific neurotransmitter polymorphism or polymorphisms, but may not have specifically endorsed that hypothesis vs. alternative hypotheses.*

Episodes of natural selection for a given gene are expected to leave characteristic signals in the DNA sequences for that gene and surrounding portions of the genome, and a variety of analytical and statistical procedures have been developed to detect these signals (Oleksyk et al., 2010; Vitti et al., 2013). There is a growing body of research applying these procedures either specifically to the genetic sequences for human neurotransmitter genes, or across the human genome as a whole (**Table 3** and references below). In this paper, we review this literature, evaluating the extent to which molecular evidence supports the various claims that natural selection has acted to promote genetic variation for human neurotransmitter genes.

**Table 3:** Recent human genome scans for natural selection.

Scan	Type of selection examined	Populations	Genotyping	Candidates Reported	Neurotransmitter gene candidates
Amorim et al., 2015	Positive	4 African and 3 American populations	Known variants	All SNPs meeting statistical threshold (*n* = 399)	None
Andrés et al., 2009	Balancing	African Americans and European Americans	Exon sequencing of subset of genome	All genes meeting statistical threshold (*n* = 60)	None
Chen et al., 2015	Positive	CEU, ASN,YRI	Known variants	20 regions with strongest signal in each population	***SLC6A4*** in ASN
Colonna et al., 2014	Positive, balancing	10 populations from Europe, Africa and East Asia	Genome sequencing	All variants meeting statistical threshold (positive: *n* = 1137, balancing: *n* = 236)	None
DeGiorgio et al., 2014	Balancing	YRI, CEU	Genome sequencing	100 strongest signals in each population	None
Fagny et al., 2014	Positive	6 populations from Europe, Africa and East Asia	Genome sequencing	100 kb windows in the 99th percentile of strongest signals for each population	None
Gompert and Buerkle, 2011	Positive, Balancing	33 populations	Known variants	Outlier regions (positive: *n* = 518; balancing: *n* = 51)	None
Grossman et al., 2013	Positive	CEU, YRI, ASN	Genome sequencing	All regions meeting statistical threshold (*n* = 412 identified in all)	None
Leffler et al., 2013	Balancing	YRI	Genome sequencing	All regions meting criterion (2 nearby shared SNPS for humans and chimps) (*n* = 125)	***HTR2A***
Liu et al., 2013	Positive	14 populations	Known variants	All regions meeting statistical criteria (*n* = 405)	***HTR2A*** in TSI, CEU ***SLC6A4*** in MAS,MEX
Lopez Herraez et al., 2009	Positive	41 populations	Known variants	All regions meeting statistical criteria (*n* = 1070, counting regions multiple times if shared among populations) (*n* = 632 unique regions)	None
Mizuno et al., 2010	Positive	YRI, CEU	Known variants	Strongest 0.1% of signals (*n* = 150–170 per population)	None
Pickrell et al., 2009	Positive	53 populations	Known variants	Top 1% of signals (*n* = 101 per ethnogeographic group)	***DRD3*, *DRD4*** in Biaka pygmies
Qian et al., 2015	Positive	YRI, CEU, ASN	Genome sequencing	All SNPs meeting statistical threshold (*n* = 6779 counting regions multiple times if shared among populations)	None
Rafajlović et al., 2014	Positive	8 populations from Africa, Europe and Asia	Genome sequencing	All regions meeting statistical criteria (*n* = 4–12 per population per method)	None
Zhou et al., 2015	Positive, balancing	YRI, CEU, CHB	Genome sequencing	All regions meeting statistical criteria (498–2960 per population for balancing; 117–485 per population for positive selection)	***SLC6A4*** in CEU,CHB (positive selection)


*Genes of the dopaminergic and serotonergic systems listed in **Table 1** are indicated if identified by scan as a possible candidate of natural selection. ASN, East Asians (Chinese and Japanese); CEU, Central and Western European descent in Utah; CHB, Han Chinese in Beijing; MAS, Malay; MEX, Mexico City; TSI, Toscani, Italia; YRI, Yoruba.*

We will first consider some of the hypotheses that have been developed implicating selection processes in the evolution of human neurotransmitter polymorphisms. Throughout this paper we focus on the genes involved in the dopaminergic and serotonergic systems that have received the bulk of attention in the literature (**Table 1**). We next briefly consider the types of molecular evidence available to address questions of selection in these genes. We then focus on presenting and evaluating the results of various molecular population genetics/genomics studies that bear on the question of selection promoting polymorphism in these genes. In the final section, we both summarize our findings, and discuss alternate explanations of the evolutionary history of the polymorphisms in these genes.

## Explanations Proposed for Polymorphisms in Human Neurotransmitter Genes

### Balancing Selection

It has frequently been suggested that balancing selection has been an important component of the processes that have produced variation in human neurotransmitter genes. Balancing selection can include a number of distinct processes in which natural selection acts to promote and maintain polymorphism (**Table 2**), including heterozygote advantage, negative frequency-dependent selection, and heterogeneity in selective advantage across time or space (Charlesworth, 2006; Hedrick, 2012; Connallon and Clark, 2013).

A few studies have found suggestions of advantageous phenotypes for heterozygotes for neurotransmitter polymorphisms. For example, Costas et al. (2011) found a small protective effect against schizophrenia for heterozygotes of the *COMT* val158met polymorphism (rs4680) in a European and West Asian sample. Gosso et al. (2008) found for this same polymorphism that Val/Met heterozygotes had superior performance in working memory tasks than either Val/Val or Met/Met homozygotes.

Negative-frequency dependent selection occurs in situations in which phenotypes (and the alleles responsible for them) are favored by virtue of being uncommon. Theory suggests that such a situation may arise for behavioral variation in populations of interacting and/or competing animals, for example, with the well-known Hawk-Dove game-theory model (Maynard Smith, 1982; Wolf and McNamara, 2012). Similarly, adopting a foraging strategy that is uncommon in a population may reap rewards due to decreased competition (Wolf and McNamara, 2012). There is evidence that in some animal species, including primates, social rank may be associated with allelic variation in neurotransmitter genes (Miller-Butterworth et al., 2008; van der Kooij and Sandi, 2015), and some authors have suggested that variation in genes underlying human behavioral differences (including neurotransmitter genes) may have evolved due to negative-frequency dependent selection arising from social and competitive interactions (Ding et al., 2002; Penke et al., 2007).

The type of balancing selection most often invoked in explanations of human behavioral and neurotransmitter variation is variation in selective pressures under different circumstances. For example, Stein et al. (2006) speculated that the *COMT* val158met polymorphism may be associated with the alternative behavioral strategies of “Warrior” (able to function well in threatening circumstances) and “Worrier” (able to perform tasks involving memory and attention in complex situations). Similarly, Garcia et al. (2010) presented evidence that alleles of *DRD4* with higher numbers of repeats in the exon III VNTR were associated with sexual promiscuity and infidelity. They argued that the *DRD4* polymorphism may have resulted from selection in some environments favoring stable sexual relationships (a “dad” strategy) while other environments have favored taking advantage of sexual opportunities, even at the expense of stable relationships (a “cad” strategy).

Selective advantage for different phenotypes (and genotypes) in different environments is also central to the differential susceptibility model of predisposition to psychiatric disorders, as well as to related concepts such as biological sensitivity to context theory (Ellis et al., 2011). These models posit that individuals differ in their sensitivity to the environments in which they develop, particularly during childhood. Some individuals are highly sensitive to the environment, particularly the social environment. Given a supportive environment in which to develop, they will flourish; given a stressful environment, they will suffer (for example with psychiatric disorders). Other individuals are less sensitive to developmental context. They will tend to neither flourish in positive environments to the same extent as the highly sensitive individuals, nor suffer as much in negative environments (Ellis et al., 2011; Belsky et al., 2015).

Differential susceptibility of this type does not necessarily imply a genetic basis for differences among individuals in environmental sensitivity (Belsky and Pluess, 2009; Frankenhuis et al., 2015). However, many studies have examined differential susceptibility in the context of particular polymorphisms, particularly for genes of the dopaminergic and serotonergic systems, including *SLC6A4*, *HTR2A*, *TPH1*, *DRD2*, *DRD4*, *COMT*, *SLC6A3*, and *MAOA* (Bakermans-Kranenburg and van IJzendoorn, 2011; Jiang et al., 2013; Belsky et al., 2015). Some authors have suggested a relatively straightforward model of the evolution of differential susceptibility for neurotransmitter genes arising from selection for different alleles under different environment context (e.g., Homberg and Lesch, 2011; Sulik et al., 2015). More highly-elaborated versions of the differential susceptibility model see different strategies evolving not just because different individuals will have superior outcomes under different circumstances, but because parents will be selected to hedge their bets by producing children who vary in their sensitivities to environmental context (Ellis et al., 2011; Frankenhuis et al., 2015).

### Positive Selection

Partially completed episodes of positive selection will be transiently associated with genetic variation in a population, as the novel allele has not yet fully replaced the ancestral allele. Polymorphisms, such as those observed for neurotransmitter genes, may therefore be the result of such partially completed processes of positive selection. Di Rienzo and Hudson (2005) proposed the ancestral susceptibility model for common human disorders with a genetic component. Alleles that were formally selected for under ancestral conditions, may now be selected against in favor of newly derived alleles in response to the profound and relatively recent changes in human diet and behavior. Carrera et al. (2009) suggested that alleles of *MAOB* associated with schizophrenia risk may be an example of the ancestral susceptibility model. Arcos-Burgos and Acosta (2007) suggested a similar explanation for allelic variation in two genes of the dopaminergic system associated with Attention Deficit Hyperactivity Disorder, *DRD4* and *SLC6A3*.

### Non-selective Processes Generating Variation

In addition to the various forms of natural selection, non-selective evolutionary processes (i.e., mutation, genetic drift) contribute to genetic variation in populations, including for neurotransmitter genes and other genes affecting human behavior and susceptibility to psychiatric disorders (Keller and Miller, 2006; Dudley et al., 2012; Verweij et al., 2012). In practice, these selectively-neutral processes are often used as a null hypothesis in tests to identify traces of selection in population genetic sequence data. Observed patterns of genetic variation in a gene or chromosome region (such as linkage disequilibrium or patterns in the allele frequency spectra) are regarded as indicating selection when these patterns appear unlikely to have resulted from neutral processes alone (Oleksyk et al., 2010; Vitti et al., 2013).

## Molecular Evidence of Selection in Human Neurotransmitter Genes

### Footprints of Positive and Balancing Selection

Episodes of natural selection acting on a genetic variant leave characteristic footprints in the DNA sequences within the population in which selection has occurred. Positive selection increases not only the frequency of the particular genetic variant under selection (for example a nucleotide substitution that causes a favorable change in the amino acid sequence of a protein) but also the frequency of whatever nearby, genetically-linked variants happen to be present on the individual chromosome in which the favorable new mutation first appeared (Vitti et al., 2013). This effect of a selective event on the genomic neighborhood of the favored variant is manifested by discernable signatures, including, for positive selection, patterns of strong linkage disequilibrium among variants in the selected region, and by effects on the spectrum of frequencies of the various genetic variants present in a population. A wide variety of statistical procedures has been developed to detect these signals of selection (Oleksyk et al., 2010; Vitti et al., 2013). Conversely, balancing selection leads to a population retaining multiple genetic variants for longer periods of time than would be expected under strictly neutral processes. This leaves footprints of balancing selection that can be distinct both from positive selection and from neutral processes: deep coalescence times for gene genealogies, characteristic allele frequency spectra, with alleles maintained at intermediate frequencies, or even polymorphisms shared among species that predate their speciation (Key et al., 2014; Fijarczyk and Babik, 2015; Gao et al., 2015).

These characteristic signatures of balancing selection are not expected to be evident until a gene has been undergoing balancing selection for a considerable period of time, at least 2N_e_ generations, with N_e_ the effective population size. Effective population sizes may be considerably less than the current census population, particularly in species that have undergone severe bottlenecks in their history (Charlesworth, 2006; Connallon and Clark, 2013). For the human species as a whole, *N*_e_ is typically estimated at a bit in excess of 10,000 (Tishkoff and Verrelli, 2003; Wall, 2003; Charlesworth, 2009). Assuming an average generation time of 25 years, this suggests for humans that the distinctive footprints of balancing selection will only be expected for polymorphisms that have been balanced for more than 500,000 years.

The footprints of more recently- initiated balancing selection may instead closely resemble those of positive selection (Andrés et al., 2009; Hedrick, 2012). Initially, a novel mutant allele that is subject to balancing selection will be below its balanced equilibrium frequency, and will increase in frequency due to selection, at the expense of the established allele, just as with positive selection.

For this reason, molecular evidence of apparent recent positive selection that has resulted in a polymorphism may possibly indicate the early stages of balancing selection. Below we review molecular evidence for neurotransmitter genes of both long-term balancing selection, and for positive selection associated with polymorphisms. We do not consider cases of selection on human neurotransmitter genes that have resulted in fixation of a new allele in the human species, for example the observation by Andrés et al. (2004) of a fixed difference in the amino acid sequence of *MAOA* between chimpanzees and humans.

### Sources of Evidence

Considering the wide-spread interest in the possibility of selection acting to maintain variation in human neurotransmitter genes (e.g., **Table 2**), there have been relatively few studies that have focused on examining molecular evidence for selection in these genes. In addition to reviewing such studies (below), we have therefore sought additional evidence through examining the results of recent studies that have scanned the entire genome for evidence of selection (**Table 3**). These genome scan studies may have a number of disadvantages compared to focused studies of the evolution of a particular gene. Many of these studies have genotyped only a particular set of previously known variant sites in the genome (such as those scans using data from the Hap-Map project), thereby creating biases in the data sets (Scheinfeldt and Tishkoff, 2013). Other scans have used obtained data from next-generation sequencing projects, often with relatively low read depths (such as Nielsen et al., 2011; 1000 Genomes Project Consortium et al., 2015). These methods are prone to sequencing errors at rates that, although not terribly great, (1000 Genomes Project Consortium et al., 2015) are nonetheless greater than for the traditional dideoxy sequencing methods often employed in single-gene studies (DePristo et al., 2011; Nielsen et al., 2011). In addition, where a study that is focused on one or a few genes may employ multiple analytical techniques for examining those sequences (e.g., Claw et al., 2010), genome scans often employ only a single statistical methodology, applying it across the entire genome (though see Grossman et al., 2013).

Because genome scans (and all molecular studies of the footprints of selection) may be prone to both false positive and negative results, various authors have suggested focusing attention on the subset of genes or genome regions identified by multiple genome-scan methods or studies (Akey, 2009; Andrés et al., 2009). There is some concordance among various studies, with the overlap among scans in the genomic regions indicated as under selection far stronger than would be expected by chance (Colonna et al., 2014). Akey (2009) reviewed the genome scans for positive selection that had been published up to that time. Focusing on eight studies that had used similar datasets, he found considerable differences among studies in the genomic regions identified as under selection, but also identified 723 genomic regions that had been identified as candidates for selection in two or more of the studies. None of these regions included any of the neurotransmitter genes that are the focus of the present review (**Table 1**).

For the current review we have examined genome scans published since Akey (2009), to see whether our genes of interest were identified as candidates of selection (**Table 3**). Many of these more recent genome scans utilize more substantial datasets (e.g., Fagny et al., 2014), and newly-developed statistical approaches (e.g., Liu et al., 2013; Colonna et al., 2014; DeGiorgio et al., 2014), and should therefore have greater power to detect selective events than the earlier scans reviewed by Akey (2009). Many of the newer scans have also used whole-genome sequencing data, while older scans typically only utilized data from projects that genotyped specific known SNPs (**Table 3**, column 3).

Some of the genome scans report genomic regions containing putative signals of natural selection, which may contain several genes. In **Table 3** we have considered a gene as having a selective signal whenever a region that includes the gene was identified by a particular genome scan. This may, of course, create false positives for the various genes in the region that were not the locus of selection. The various genome scan papers also differ considerably in their reporting of genes identified as candidates of selection. Some report every SNP, gene or genomic region that meets a particular statistical criterion, while others report only a relatively small number of the most extreme signals (**Table 3**, column 4). If a scan has reported numerous candidates for selection, but not identified any of the neurotransmitter genes (e.g., Qian et al., 2015), this may strengthen the case that these genes do not exhibit strong signs of having been under selection. Conversely, if a study has reported only a very few of the strongest signals of selection, but this includes one of our neurotransmitter genes (e.g., Chen et al., 2015) that may provide a particularly strong indication of selection on that gene or genomic region.

### Claims of Molecular Evidence of Natural Selection in Human Neurotransmitter Genes

#### Balancing Selection

Several genome scans studies have focused on detecting signals of balancing selection in human evolution (e.g., Andrés et al., 2009; Leffler et al., 2013; DeGiorgio et al., 2014). Leffler et al. (2013) looked for evidence of very long-term balancing selection through polymorphisms shared in both humans and common chimpanzees. Only two of the polymorphic SNPs they identified were associated with human neurotransmitter genes, both with the upstream (promoter) region of the serotonin receptor *HTR2A*. However, both of these polymorphisms (rs6311 and rs36213156) are C/T polymorphisms associated with CpG dinucleotides, that is sites with a C and a G nucleotide adjacent in a DNA strand. Such sites are associated with a particularly high rate of mutation, particularly transitions such as a change of the cytosine residue to a thymine (Nachman and Crowell, 2000). This increases the likelihood that the observed polymorphisms are shared between humans and chimpanzees due to recurrent mutation, rather than by balancing selection that has been maintained since prior to speciation (Leffler et al., 2013).

DeGiorgio et al. (2014) applied a novel gene-genealogy approach to scan for balancing selection across the human genome. They identified a number of regions of the genome with apparent signs of balancing selection. These regions included many genes associated with immune function, as well as with cell membranes, cell adhesion and cell junctions. None of the candidate regions of balancing selection were associated with neurotransmitter genes.

Andrés et al. (2009), starting with a set of 13,400 human genes, examined in detail the 4,877 genes that contained either polymorphisms or fixed differences between humans and chimpanzees. They identified 60 candidate genes with signals of balancing selection. As with DeGiorgio et al. (2014) a high proportion of the associated genes were associated with immune function, but none were associated with neurotransmitter function.

Several additional genome scans were designed to detect signals of either positive or balancing selection (Gompert and Buerkle, 2011; Colonna et al., 2014; Zhou et al., 2015). Each scan identified a number of candidates for balancing selection, however, none of these candidate regions included our neurotransmitter genes of interest.

Along with these genome-scan approaches, a number of studies have focused on examining specific neurotransmitter genes for evidence of selection. Perhaps the strongest claims that some form of balancing selection has occurred are for the dopamine receptor genes *DRD4* and *DRD2*. Ding et al. (2002) observed that a strikingly high proportion of the SNPs in the exons of *DRD4* cause amino acid changes (rather than being synonymous substitutions). This pattern suggests selection acting in favor of changes in the protein (Kreitman, 2000), perhaps indicating negative-frequency dependent selection on this gene. Conceivably, such selection may be wide-spread among primates. Livak et al. (1995) observed a very high degree of amino acid sequence diversity in *DRD4* across a range of ape and monkey species (both old-world and new-world). However, none of the genome scan studies we examined detected a statistical signal of such very long-term balancing selection in *DRD4* (**Table 3**), nor have several other studies that have examined human *DRD4* variation (Wang et al., 2004; Hattori et al., 2009; Naka et al., 2011). It may be that the unusual degree of amino acid divergence has somehow emerged from the properties of *DRD4* that give it a particularly high rate of mutation, including the presence of a tandem repeat in the coding sequence associated with a high rate of unequal recombination (Ding et al., 2002).

Wang et al. (2004) argued that the human allele frequency spectrum for *DRD4* showed signs of balancing selection exclusive to the human lineage, with there being a greater number of alleles of intermediate frequencies and fewer very rare alleles than expected under selective neutrality. However, Wang et al. (2004) obtained a statistically significant *negative* value when formally testing for departures from neutrality with a Tajima’s D test. Long-term balancing selection should instead be associated with a significant positive value for Tajima’s D (Tajima, 1989). The negative D is, however, consistent with the argument of Wang et al. (2004) that we may be in the early stages of balancing selection, in which the molecular signal is one of positive selection, due to the recent rapid increase in frequency of one of the balanced alleles. We reserve further discussion of this question to the section on positive selection below.

Mota et al. (2012) detected a possible signal of balancing selection in another dopamine receptor, *DRD2*. They reported than one human *DRD2* polymorphism (rs6277) is shared with Neandertals. They proposed that such long-term maintenance of the polymorphism suggests balancing selection, although they did not perform any specific tests to evaluate this supposition. Although Mota et al. (2012) did not discuss the possibility that the presence in the two species might reflect recurrent mutation, it should be noted that the polymorphism is found at a CpG site, and represents a characteristic mutation (C to T) associated with such sites. Further supporting the hypothesis of independent origins in modern humans and Neandertals, Mota et al. (2012) calculated the age of appearance of the derived allele at 359,000 years in Europeans and 65,000 years in other populations. This suggests the possibility of distinct origins for different versions of the derived (T) allele, with the European version possibly an introgression from Neandertals (Mota et al., 2012).

Göllner and Fieder (2015) looked for candidate SNPS in *DRD2* using statistical methods that examine differentiation among populations in allele frequencies (F_ST_) across a global human sample. The logic of these tests for identifying balancing selection is that an unusually high degree of similarity in allele frequencies among populations may be achieved if balancing selection is acting similarly in each population to maintain allelic diversity. They identified nine SNPS as candidates of balancing selection in *DRD2*. These did not include the rs6277 SNP discussed by Mota et al. (2012). The statistical approaches used by Göllner and Fieder (2015) may be subject to false positives under a number of circumstances (Foll and Gaggiotti, 2008; Vitalis et al., 2014). Additionally, as discussed above, none of the genome scans studies for balancing selection identified *DRD2* as a candidate. *DRD2* is therefore probably best regarded not as an established case of balancing selection, though it may be worthy of further exploration.

Overall, there is an absence of any clearly-established cases of molecular evidence for balancing selection *per se* in human neurotransmitter genes. However, as discussed above, cases of recently-initiated balancing selection may appear as signals of positive selection in genomic analyses. We therefore review next the evidence for positive selection in human neurotransmitter genes.

#### Positive Selection

The strongest evidence for positive selection in a human neurotransmitter gene is for the serotonin transporter *SLC6A4*. Claw et al. (2010) found that the population genetics of one haplotype of this gene, which they termed “S/12/G,” had a set of distinctive features. For a common haplotype (36% in their global sample), there was a significantly low degree of SNP variation compared to other haplotypes of the *SLC6A4* gene. The 36% global frequency also appears quite high (in absence of positive selection) for a haplotype with an estimated most recent common ancestor at 19 kya (Claw et al., 2010). Coalescent simulations further suggest that the patterns of SNP diversity, haplotype frequency and geographic variation in haplotype frequencies across populations could not be explained by a combination of neutral and demographic processes (Claw et al., 2010).

Crespi et al. (2007) examined 76 genes putatively associated with schizophrenia risk for evidence of positive selection. They queried the Haplotter database, representing analyses using an extended-haplotype based test (iHS) in one sample each representing African, Asian and European populations (Voight et al., 2006). Genes examined included *COMT*, *DRD2*, *DRD3*, *DRD4*, *MAOA*, *SLC6A4*, and *TPH1*. *SLC6A4* was in a region with a signal of positive selection for both the European and Asian samples. In all, 14 of the 76 genes examined showed signals of positive selection, but *SLC6A4* was the only one of our genes of interest to do so. Murdoch et al. (2013) also identified a signal of selection in the region of *SLC6A4* in the Haplotter data, although they argued that selection may have actual been for a nearby, linked gene.

Signals of positive selection in regions of the 17th chromosome that contain the *SLC6A4* gene have also been detected in three recent genome scan studies using different methodologies to detect selection (Liu et al., 2013; Chen et al., 2015; Zhou et al., 2015). Chen et al. (2015) reported this region to be among the 20 genomic regions showing the strongest signals of positive selection in an East Asian (Chinese and Japanese) sample. Liu et al. (2013) found signals of positive selection in both Malay and Mexico City samples. Zhou et al. (2015) found selection on this region in samples of Han Chinese in Beijing (CHB) and people of Northern/Western European ancestry in Utah (CEU). From rates of coalescence, they also estimated the age of the onset of selection for this genomic region as 904 generations in CHB and 988 generations in CEU. Estimating generations at 25 years, this corresponds to selection originating at ∼23–25 kya. This corresponds fairly well to the estimate of Claw et al. (2010) of the last common ancestor of extant copies of the S/12/G haplotype at 19 kya. Taken together, these results seem broadly consistent with selection favoring this haplotype beginning at around that time. The results of these studies also suggest East Asia as the center of origin and/or the center of selection for this haplotype, given that the greatest measured frequency of this haplotype is in Southeast Asia (Claw et al., 2010) and that three genome scans found evidence of selection in at least some East Asian populations. Given the finding of evidence for selection in a Mexico City sample (Zhou et al., 2015), it is also noteworthy that the highest frequency of S/12/G outside of Asia was found in a Mexican sample (Claw et al., 2010). On the other hand, it is somewhat difficult to reconcile this particular account with the reports by Zhou et al. (2015) and by Crespi et al. (2007) of selection on *SLC6A4* in CEU, considering that the S/12/G haplotype has relatively low frequency in Western Europe, and so is unlikely to have been the target of selection in that geographic region (Claw et al., 2010). It may also be difficult to reconcile a unique origin of this haplotype at 19 kya with the existence of this haplotype at frequencies greater than ∼10% in all measured populations, including in Sub-Sahara Africa (Claw et al., 2010). As Claw et al. (2010) suggest, elucidation of the evolutionary history of this genomic region may require both increased sampling and detailed modeling of selective and demographic factors.

While no other human neurotransmitter gene has as strong evidence for natural selection as *SLC6A4*, there have been reports suggesting the possibility of selection on another gene in the serotonin system, the receptor gene *HTR2A*. Similar to their findings with *SLC6A4*, Claw et al. (2010) found that one haplotype group of *HTR2A*, -1438A/102T, had significantly low SNP diversity for its frequency (40%) compared to other haplotypes of the gene. Coalescent simulations suggested that neutral and demographic processes could not explain this pattern, suggesting the possibility that selection has acted on this allele. One genome scan, Liu et al. (2013) found signals of positive selection in *HTR2A* in two European-derived samples (one from Italy, and the aforementioned CEU). However, other genome scans have not identified *HTR2A* as a candidate of selection. Overall, there is therefore only little evidence suggesting a history of selection on this gene.

Another serotonergic gene has been suggested to potentially exhibit differential susceptibility-type balancing selection: *TPH1*, which codes for one of the isoforms of the rate-limiting enzyme in serotonin production (Belsky et al., 2015). We are aware of no studies focusing on molecular evidence for selection in this gene, and it has not been identified as a candidate of selection in any of the genomic scans we examined (**Table 3**).

There have been several claims of molecular evidence for selection in human dopaminergic genes, particularly for dopamine receptor genes, with the largest amount of attention paid to *DRD4*. Two papers (Ding et al., 2002; Wang et al., 2004) reported evidence for selection on this gene, either positive selection, or the early stages of balancing selection (see above). As noted above Ding et al. (2002) observed in the exons a high K_a_/K_s_ (the ratio of the rate of variation leading to amino acid changes vs. synonymous variation), suggestive of selection on the protein amino acid sequence. In addition, the *DRD4* gene has a variable number tandem repeat (VNTR) in exon 3, with the number of repeats ranging from 2 to 11 (Ding et al., 2002). Ding et al. (2002) found that the derived 7 repeat variant (7R) was in strong linkage disequilibrium with two SNPs. They suggested that the relatively high frequency of the 7R variant (19% in their worldwide sample), coupled with the high degree of linkage disequilibrium was best explained by an origination of the 7R allele within the last 50,000 years, and subsequent selection-driven increase in its frequency. A follow-up study by the same research group confirmed this pattern of linkage disequilibrium (Wang et al., 2004). These authors additionally applied several types of allele frequency spectrum tests (Tajima’s D, Fu and Li’s D* and F*) and found departures from neutral expectations consistent with positive selection (Wang et al., 2004).

More recent studies have not supported these earlier conclusions. Naka et al. (2011) examined linkage disequilibrium over a larger region surrounding the exon 3 VNTR region than had the previous studies, and using tests that had not been developed at the time of the previous studies. They found no evidence that linkage disequilibrium was substantially greater or homozygosity adjacent to 7R variant substantially less than for other genes. Applying the Ewens-Watterson test, they also found no evidence for significant departures from neutrality in the allele frequency spectrum. Both Hattori et al. (2009) and Naka et al. (2011) also suggested that the 7R allele was older than indicated by previous studies, so that its frequency was not unusually high given its age.

One genome scan that we examined identified *DRD4* as exhibiting a signal of positive selection. Pickrell et al. (2009) found a moderately strong signal of an extended haplotype in this region in their Biaka pygmy sample. Signals of positive selection on *DRD4* have not found in other genome scans.

There has been one report of molecular evidence for selection on the dopamine receptor gene *DRD3*. Costas et al. (2009), investigated a possible association of a SNP in *DRD3* affecting amino acid sequence (Ser9Gly) with susceptibility to schizophrenia. They found that the derived Ser variant was associated in CEU with a particular haplotype multi-SNP haplotype associated with reduced risk of schizophrenia in case-control studies on European-derived populations. They also found that that this haplotype showed a pattern of extended homozygosity suggestive of positive selection.

One genome scan, (Pickrell et al., 2009) identified *DRD3* as a candidate for selection in the same African Biaka pygmy population sample identified as having signals for selection in *DRD4*. An African selection event in *DRD3* would likely not have involved positive selection on the Ser-containing haplotype seen in CEU. This haplotype is found much less commonly in Africa (∼12%) than in CEU (65%) or in East Asia (63–76%), leading Costas et al. (2009) to argue that positive selection on the Ser-containing haplotype had not occurred in Sub-Saharan Africa.

Göllner and Fieder (2015), examined the possibility of positive selection on SNPs in *DRD2*. They found no candidate SNPs that stood up to stringent analysis. Both *DRD2* and *COMT* (coding for an enzyme involved in degradation of dopamine and other catecholamines) have often been suspected as being involved in differential susceptibility to behavioral disorders or otherwise subject to balancing selection (**Table 2**). Neither of these genes was identified as a candidate of selection in any of the genome scans we examined, nor are we aware of any published studies that present molecular evidence for positive selection acting on *COMT*.

There have been two reports of evidence of positive selection acting on genes coding for monoamine oxidase enzymes, involved in degradation of various neurotransmitters, including both dopamine and serotonin. Gilad et al. (2002) sequenced portions of the *MAOA* gene for 56 males from a variety of ethnogeographic groups. They found substantial linkage disequilibrium across the gene, and argued that positive selection was a likely explanation. They also applied two frequency-spectrum tests to the data set, finding a significant value for Fay and Wu’s H, but not for Tajima’s D. For the other monoamine oxidase gene, *MAOB*, Carrera et al. (2009) found the signal of extended haplotype homozygosity in the region of this gene for CEU was in the 95th–97th percentile of all genes on the X chromosome. They also found that in a case-control study in Spain, a SNP associated with the putatively selected haplotype was associated with protection from schizophrenia in males, but not females. They suggested that the ancestral allele may have been adaptive under former conditions, but was currently being selectively replaced, as recent changes in human lifestyles had changed the selective regime. Positive selection for the monoamine oxidase genes has not been confirmed by any additional studies of which we are aware; neither was identified as a candidate for selection in any of the genome scans we analyzed.

## General Discussion

As of now, *SLC6A4* is the only gene of the dopaminergic and serotonergic systems for which multiple studies have unambiguously supported the operation of natural selection. Currently, there is very limited information available about either the precise phenotypic/behavioral traits that may have been selected for, or the biochemical/physiological aspects of function that may have been altered in alleles of this gene that have been under selection.

The S/12/G haplotype that appears to have been positively selected does not affect the structure of the resulting serotonin transporter protein, and any effects of this allele are presumably mediated through control of gene expression (Claw et al., 2010). However, it is not clear what effects on expression to anticipate with this haplotype. This haplotype includes the short (S) variant of the promoter-region VNTR, which a number of studies have found to be associated with decreased transcription and decreased serotonin reuptake activity (Iurescia et al., 2015). However, the haplotype also includes the 12-repeat variant of the Intron 2 VNTR, which has been associated with *increased* gene expression (Murdoch et al., 2013). Prediction of the level of gene expression associated with this haplotype is further complicated by studies suggested that transcription is also strongly affected by several SNPs (Iurescia et al., 2015) that were not genotyped in the Claw et al. (2010) study. In addition, Ali et al. (2010) found that expression of a gene construct combining variants in different portions of *SLC6A4* could not be predicted from the expression of each variant individually.

Reported associations of particular molecular variants in *SLC6A4* with behavioral and psychiatric phenotypes are also subject to some uncertainty. Studies have often genotyped only a single polymorphic site within the gene. However, there is substantial linkage disequilibrium among the variant sites, and reported associations of particular variants with behaviors may therefore have misidentified the polymorphisms that are functionally responsible for the behavioral effects (Claw et al., 2010; Murdoch et al., 2013). In addition, phenotypic effects of variation in *SLC6A4* may depend on interactions with other genes in the serotonergic system (Claw et al., 2010). Better understanding of the implications of the various polymorphisms in *SLC6A4* both for gene expression and for behavioral phenotypes will therefore be important steps in discovering which variants have been under selection, and elucidating the adaptive significance of the selective processes that have been occurring.

The signal that has been identified for *SLC6A4* is of positive selection, rather than balancing selection. It may be that what is seen with *SLC6A4* is the early stages of evolution of a balanced polymorphism. Nonetheless, in spite of a great deal of speculation about possible balancing selection in human neurotransmitter genes (**Table 2**) there are no cases in which molecular evidence strongly supports the operation of balancing selection for these genes (though genes with equivocal evidence such as *DRD2* and *HTR2A* may be worth further exploration).

That molecular evidence for selection has not been detected in a gene (or is detected by only a single study, while not found in others) does not, of course, definitively demonstrate that there has been no selection. There is a host of reasons why genuine episodes of selection may be missed in these studies. Selection may, for example, have been weak, or have been obscured by demographic history (Scheinfeldt and Tishkoff, 2013). However, in spite of these potential difficulties which face *any* study examining selection for *any* gene, population genomics studies have proven able to identify clear episodes of selection. For balancing selection, the MHC complex is consistently highlighted by genome scans, as are genes for a variety of cell surface proteins (Andrés et al., 2009; DeGiorgio et al., 2014). Scans for positive selection tend to detect classic cases of selection acting on genes such as *LCT*, SLC24A5, and *EDAR* (Liu et al., 2013; Scheinfeldt and Tishkoff, 2013). If we cannot definitely say that there has not been recent selection on the various neurotransmitter genes that have attracted the most interest (**Table 1**), it seems to say based on current evidence that these genes (with the possible exception of *SLC6A4*) are not among the more compelling known cases of either positive or balancing selection.

The lack of clear signals of balancing selection in human neurotransmitter genes is broadly consistent with arguments that polygenic variation for human behavioral/mental health traits has largely not involved balancing selection. Verweij et al. (2012) found that only a small proportion of genetic variation in human personality traits was associated with polymorphic loci of the type we have been considering in this paper. Most variation instead appeared to be associated with rare genetic variants at a large number of loci. They suggested that this polygenic variation was likely due largely to selection-mutation balance, rather than balancing selection. Keller and Miller (2006) similarly considered the possibility of balancing selection for susceptibility to mental disorders, but concluded that polygenic mutation-selection balance was likely to most responsible for observed genetic susceptibility.

Mutation-selection balance will typically maintain an allele at only fairly low frequencies in a population. If non-selective processes are responsible for the development of the polymorphisms seen in human neurotransmitter genes, this has likely involved processes in addition to mutation. Certainly, neutral processes such as genetic drift must have an important role in the development of human polymorphisms in general. The human genome contains approximately eight million genetic variants with minor allele frequencies greater than 5% (1000 Genomes Project Consortium et al., 2015). It is implausible that variation in so many sites could be maintained by selection. Indeed, models of neutral processes suggest that the great majority of observed polymorphic genetic variants will have reached high-frequency due to neutral processes rather than selection (Dudley et al., 2012).

Selective neutrality for genes that affect behavior might arise from tradeoffs faced with various behavioral strategies, each with advantages and disadvantages, such that on average, no one variant is favored (MacDonald, 1995; Nettle, 2006). For example, greater conscientiousness may be associated with behaviors promoting health and the ability to achieve long-term payoffs, but at the expense of not taking advantage of opportunities for receiving immediate rewards, including short-term mating relationships (Nettle, 2006). It may be that for any dimension of behavioral and personality variation, fitness is approximately equal across the normal range of variation (MacDonald, 1995; Nettle, 2006). Similarly, tradeoffs may exist for alleles that lead to susceptibility for psychiatric disorders. For example, alleles associated with schizophrenia in some individuals may in other individuals be associated with creativity, problem-solving, or other cognitive functions (Nettle, 2006; Crespi et al., 2007; Crespi, 2009).

The concept of tradeoffs for behavioral/genetic variation shares with the concept of balancing selection under environmental heterogeneity the conjecture that different behaviors/phenotypes/strategies may be most successful under different circumstances. With either concept, there is no universally favored strategy, and natural selection does not lead to fixation of a single trait. There are, however, important distinctions between the two concepts. Modeling suggests that for selective heterogeneity to result in balancing selection, there must be subdivision of a population into discrete spatial patches with differing selective regimes (Hedrick, 2006; Svardal et al., 2015). In absence of this condition, selection will simply favor whichever trait has the highest weighted average fitness across the selective conditions that individuals within the population face (Keller and Miller, 2006). Most importantly, these concepts differ in their predictions of the evolutionary dynamics of the traits in question. With balancing selection, particular allele frequencies are maintained in a population, assuming that the selective regime remains stable. Similarly, different populations subject to similar selective regimes will be expected to converge on allele frequencies. Conversely, if tradeoffs simply lead to approximately equal average fitness for different phenotypes/genotypes, alleles will be selectively near-neutral. Allele frequencies will drift in frequency over time in any one population, and may come to differ markedly among populations, even though the selective regime does not appreciably differ between them.

Arguing against selective neutrality for genes affecting human behavior and personality, Keller and Miller (2006) calculated that with a human effective population size of ∼10,000, alleles would be selectively near-neutral only if individuals with the least fit genotype had average reproductive success at least 99.997% of those with the most fit genotype. Keller and Miller (2006) and Penke et al. (2007) argued that many genetic variants that affect behavior and the probability of developing psychiatric disorders would surely have average effects on fitness stronger than this. This evolutionary trajectory of these variants would thus be governed more by selection than by genetic drift.

One factor to consider in assessing the plausibility of a neutral explanation for human polymorphisms is that the effective human population size of any individual geographic region may be a good deal smaller than 10,000. Human populations outside of Africa appear to have undergone bottlenecks between 15,000 and 20,000 years ago with *N*_e_ less than 1500 for long periods of time (1000 Genomes Project Consortium et al., 2015). Even this may overstate the relevant population sizes, due to the human history of geographic spread across the globe from an initial distribution in Africa (Tishkoff and Verrelli, 2003). As a species spreads across a landscape by a succession of founder events, allele frequencies may be highly subject to a selectively-neutral “surfing” phenomenon analogous to genetic drift. The relevant “effective propagule size” governing changes in allele frequencies may be a good deal smaller than standardly-calculated effective population sizes (Slatkin and Excoffier, 2012). This surfing effect may also lead to pronounced geographic variation in allele frequencies. For example, surfing during the Paleolithic human expansion into Europe is a possible explanation for east–west clines in allele frequency in that region (Klopfstein et al., 2006).

The tendency of serial founder effects to lead to altering allele frequencies as waves of population expansion spread may explain the pronounced geographic differences in the frequencies of neurotransmitter polymorphisms. For example, the derived A variant of the *DRD2* rs6277 polymorphism is found at less than 5% in most African samples, and shows an east–west cline in Eurasia, with frequencies in east Asian typically 5–10%, in

Europe 40–60%, and in central Asia intermediate between those values. A very similar geographic pattern is seen for the derived A variant in the *COMT* rs4680 SNP, and pronounced geographic variation also exists for the frequencies of other prominent neurotransmitter gene polymorphisms such as the *DRD4* exon 3 VNTR and the rs6280 missense SNP in *DRD3*(data from ALFRED, accessed November 2015).

While the extensive geographic variation for neurotransmitter genes is compatible with drift/surfing as the explanation for most of the polymorphisms, selective explanations for the polymorphisms require the assumption that selective regimes have been very different in different parts of the world. One case in which a functional interpretation has been given to geographic patterns of variation in a neurotransmitter polymorphism is with the *DRD4* Exon 3 VNTR. Matthews and Butler (2011) presented evidence that the frequencies of the derived 2R and 7R variants increase with migration distance out of Africa. They argued that these variants are associated with novelty-seeking personalities, and that the association of these alleles with migratory distance from Africa may have resulted from either selection for the ability to adapt to novel environments encountered during migration, or from an enhanced tendency in carriers of 2R and 7R to seek out novel environments by migration.

Selective explanations for the geographic variation in the various neurotransmitter polymorphisms, even if based on plausible or demonstrable phenotypic associations of the genetic variants, face the difficulty that, as detailed above, molecular tests do not generally support these genes as targets of selection. In this context it is worth noting that a number of the genome scans we examined were designed to detect differences among populations in selective history, but did not identify the neurotransmitter genes as candidates of selection (e.g., Mizuno et al., 2010; Gompert and Buerkle, 2011; Colonna et al., 2014; Amorim et al., 2015).

The preponderance of evidence from population genomics studies suggests that natural selection has not been a particularly important factor in the evolution of polymorphisms for most genes of the dopaminergic and serotonergic systems. This^1^ does not directly bear on questions about the functional and phenotypic consequences of these polymorphisms. The importance of variation in these genes for understanding human differences and the etiology of mental health conditions depends on whether the variants are reliably associated with particular behaviors or conditions. Although some reported associations of alleles of these genes with behaviors and mental health status have not withstood attempts at replication, other findings appear more robust (e.g., Balestri et al., 2014; Gatt et al., 2015). Variation in dopaminergic and serotonergic genes may also have important clinical significance as predictors of patient responses to psychiatric drugs (Lally and MacCabe, 2016). Again, the utility of molecular variants in this regard does not depend on the evolutionary processes that have produced the polymorphisms. What studies of the selective history of these genes do is to inform us about the processes by which genetic variation for these characters has been generated. Whatever the current functional importance of the different alleles for these genes, the evidence from population genomics analyses suggests that the observed genetic variation may have resulted largely from selectively-neutral processes.

## Author Contributions

DT and JP developed the questions and identified, analyzed and synthesized available literature. DT wrote the manuscript with input from JP.

## Conflict of Interest Statement

The authors declare that the research was conducted in the absence of any commercial or financial relationships that could be construed as a potential conflict of interest.

## Footnotes

1 http://alfred.med.yale.edu

## References

[B1] 1000 Genomes Project Consortium Auton A. Brooks L. D. Durbin R. M. Garrison E. P. Kang H. M. (2015). A global reference for human genetic variation. *Nature* 526 68–74. 26432245 10.1038/nature15393 PMC4750478

[B2] Akey J. M. (2009). Constructing genomic maps of positive selection in humans: where do we go from here? *Genome Res.* 19 711–722. 10.1101/gr.086652.108 19411596 PMC3647533

[B3] Ali F. R. Vasiliou S. A. Haddley K. Paredes U. M. Roberts J. C. Miyajima F. (2010). Combinatorial interaction between two human serotonin transporter gene variable number tandem repeats and their regulation by CTCF. *J. Neurochem.* 112 296–306. 10.1111/j.1471-4159.2009.06453.x 19860858 PMC2848977

[B4] Amorim C. E. Daub J. T. Salzano F. M. Foll M. Excoffier L. (2015). Detection of convergent genome-wide signals of adaptation to tropical forests in humans. *PLoS ONE* 10:e0121557 10.1371/journal.pone.0121557 PMC4388690 25849546

[B5] Andrés A. M. Hubisz M. J. Indap A. Torgerson D. G. Degenhardt J. D. Boyko A. R. (2009). Targets of balancing selection in the human genome. *Mol. Biol. Evol.* 26 2755–2764. 10.1093/molbev/msp190 19713326 PMC2782326

[B6] Andrés A. M. Soldevila M. Navarro A. Kidd K. K. Oliva B. Bertranpetit J. (2004). Positive selection in MAOA gene is human exclusive: determination of the putative amino acid change selected in the human lineage. *Hum. Genet.* 115 377–386. 15349769 10.1007/s00439-004-1179-6

[B7] Arcos-Burgos M. Acosta M. T. (2007). Tuning major gene variants conditioning human behavior: the anachronism of ADHD. *Curr. Opin. Genet. Dev.* 17 234–238. 10.1016/j.gde.2007.04.011 17467976

[B8] Bakermans-Kranenburg M. J. van IJzendoorn M. H. (2011). Differential susceptibility to rearing environment depending on dopamine-related genes: new evidence and a meta-analysis. *Dev. Psychopathol.* 23 39–52. 10.1017/S0954579410000635 21262038

[B9] Balestri M. Calati R. Serretti A. De Ronchi D. (2014). Genetic modulation of personality traits: a systematic review of the literature. *Int. Clin. Psychopharmacol.* 29 1–15. 10.1097/YIC.0b013e328364590b 24100617

[B10] Belsky J. Newman D. A. Widaman K. F. Rodkin P. Pluess M. Fraley R. C. (2015). Differential susceptibility to effects of maternal sensitivity? A study of candidate plasticity genes. *Dev. Psychopathol.* 27 725–746. 10.1017/S0954579414000844 25221946

[B11] Belsky J. Pluess M. (2009). Beyond diathesis stress: differential susceptibility to environmental influences. *Psychol. Bull.* 135 885–908. 10.1037/a0017376 19883141

[B13] Carrera N. Sanjuan J. Molto M. D. Carracedo A. Costas J. (2009). Recent adaptive selection at MAOB and ancestral susceptibility to schizophrenia. *Am. J. Med. Genet. B Neuropsychiatr. Genet.* 150b, 369–374. 10.1002/ajmg.b.30823 18553363

[B14] Charlesworth B. (2009). Fundamental concepts in genetics: effective population size and patterns of molecular evolution and variation. *Nat. Rev. Genet.* 10 195–205. 10.1038/nrg2526 19204717

[B15] Charlesworth D. (2006). Balancing selection and its effects on sequences in nearby genome regions. *PLoS Genet.* 2:e64 10.1371/journal.pgen.0020064 PMC1449905 16683038

[B16] Chen H. Hey J. Slatkin M. (2015). A hidden Markov model for investigating recent positive selection through haplotype structure. *Theor. Popul. Biol.* 99 18–30. 10.1016/j.tpb.2014.11.001 25446961 PMC4277924

[B17] Claw K. G. Tito R. Y. Stone A. C. Verrelli B. C. (2010). Haplotype structure and divergence at human and chimpanzee serotonin transporter and receptor genes: implications for behavioral disorder association analyses. *Mol. Biol. Evol.* 27 1518–1529. 10.1093/molbev/msq030 20118193 PMC2912469

[B18] Colonna V. Ayub Q. Chen Y. Pagani L. Luisi P. Pybus M. (2014). Human genomic regions with exceptionally high levels of population differentiation identified from 911 whole-genome sequences. *Genome Biol.* 15:R88 10.1186/gb-2014-15-6-r88 PMC4197830 24980144

[B19] Connallon T. Clark A. G. (2013). Antagonistic versus nonantagonistic models of balancing selection: characterizing the relative timescales and hitchhiking effects of partial selective sweeps. *Evolution* 67 908–917. 10.1111/j.1558-5646.2012.01800.x 23461340 PMC3590853

[B20] Costas J. Carrera N. Dominguez E. Vilella E. Martorell L. Valero J. (2009). A common haplotype of DRD3 affected by recent positive selection is associated with protection from schizophrenia. *Hum. Genet.* 124 607–613. 10.1007/s00439-008-0584-7 18987889

[B21] Costas J. Sanjuan J. Ramos-Rios R. Paz E. Agra S. Ivorra J. L. (2011). Heterozygosity at catechol-O-methyltransferase Val158Met and schizophrenia: new data and meta-analysis. *J. Psychiatr. Res.* 45 7–14. 10.1016/j.jpsychires.2010.04.021 20488458

[B22] Crespi B. (2009). “Evolutionary genetics of affective disorders,” in *Understanding Depression: A Translational Approach*, eds Pariant C. Nesse R. M. Nutt D. Wolpert L. (Oxford: Oxford University Press), 37–54.

[B23] Crespi B. Summers K. Dorus S. (2007). Adaptive evolution of genes underlying schizophrenia. *Proc. Biol. Sci.* 274 2801–2810. 10.1098/rspb.2007.0876 17785269 PMC2288689

[B24] DeGiorgio M. Lohmueller K. E. Nielsen R. (2014). A model-based approach for identifying signatures of ancient balancing selection in genetic data. *PLoS Genet.* 10:e1004561 10.1371/journal.pgen.1004561 PMC4140648 25144706

[B25] DePristo M. A. Banks E. Poplin R. Garimella K. V. Maguire J. R. Hartl C. (2011). A framework for variation discovery and genotyping using next-generation DNA sequencing data. *Nat. Genet.* 43 491–498. 10.1038/ng.806 21478889 PMC3083463

[B26] Di Rienzo A. Hudson R. R. (2005). An evolutionary framework for common diseases: the ancestral-susceptibility model. *Trends Genet.* 21 596–601. 10.1016/j.tig.2005.08.007 16153740

[B27] Ding Y. C. Chi H. C. Grady D. L. Morishima A. Kidd J. R. Kidd K. K. (2002). Evidence of positive selection acting at the human dopamine receptor D4 gene locus. *Proc. Natl. Acad. Sci. U.S.A.* 99 309–314. 10.1073/pnas.012464099 11756666 PMC117557

[B28] Dudley J. T. Kim Y. Liu L. Markov G. J. Gerold K. Chen R. (2012). Human genomic disease variants: a neutral evolutionary explanation. *Genome Res.* 22 1383–1394. 10.1101/gr.133702.111 22665443 PMC3409252

[B29] Ebstein R. P. Israel S. Chew S. H. Zhong S. Knafo A. (2010). Genetics of human social behavior. *Neuron* 65 831–844. 10.1016/j.neuron.2010.02.020 20346758

[B30] Ellis B. J. Boyce W. T. Belsky J. Bakermans-Kranenburg M. J. van IJzendoorn M. H. (2011). Differential susceptibility to the environment: an evolutionary–neurodevelopmental theory. *Dev. Psychopathol.* 23 7–28. 10.1017/S0954579410000611 21262036

[B31] Fagny M. Patin E. Enard D. Barreiro L. B. Quintana-Murci L. Laval G. (2014). Exploring the occurrence of classic selective sweeps in humans using whole-genome sequencing data sets. *Mol. Biol. Evol.* 31 1850–1868. 10.1093/molbev/msu118 24694833

[B32] Fijarczyk A. Babik W. (2015). Detecting balancing selection in genomes: limits and prospects. *Mol. Ecol.* 24 3529–3545. 10.1111/mec.13226 25943689

[B33] Foll M. Gaggiotti O. (2008). A genome-scan method to identify selected loci appropriate for both dominant and codominant markers: a Bayesian perspective. *Genetics* 180 977–993. 10.1534/genetics.108.092221 18780740 PMC2567396

[B34] Frankenhuis W. E. Panchanathan K. Belsky J. (2015). A mathematical model of the evolution of individual differences in developmental plasticity arising through parental bet-hedging. *Dev. Sci.* 19 251–274. 10.1111/desc.12309 26010335

[B35] Gao Z. Przeworski M. Sella G. (2015). Footprints of ancient-balanced polymorphisms in genetic variation data from closely related species. *Evolution* 69 431–446. 10.1111/evo.12567 25403856 PMC4335603

[B36] Garcia J. R. MacKillop J. Aller E. L. Merriwether A. M. Wilson D. S. Lum J. K. (2010). Associations between dopamine D4 receptor gene variation with both infidelity and sexual promiscuity. *PLoS ONE* 5:e14162 10.1371/journal.pone.0014162 PMC2994774 21152404

[B37] Gatt J. M. Burton K. L. Williams L. M. Schofield P. R. (2015). Specific and common genes implicated across major mental disorders: a review of meta-analysis studies. *J. Psychiatr. Res.* 60 1–13. 10.1016/j.jpsychires.2014.09.014 25287955

[B38] Gilad Y. Rosenberg S. Przeworski M. Lancet D. Skorecki K. (2002). Evidence for positive selection and population structure at the human MAO-A gene. *Proc. Natl. Acad. Sci. U.S.A.* 99 862–867. 10.1073/pnas.022614799 11805333 PMC117396

[B39] Göllner T. Fieder M. (2015). Selection in the dopamine receptor 2 gene: a candidate SNP study. *PeerJ* 3:e1149 10.7717/peerj.1149 PMC4540012 26290802

[B40] Gompert Z. Buerkle C. A. (2011). A hierarchical Bayesian model for next-generation population genomics. *Genetics* 187 903–917. 10.1534/genetics.110.124693 21212231 PMC3063681

[B41] Gosso M. F. de Geus E. J. Polderman T. J. Boomsma D. I. Heutink P. Posthuma D. (2008). Catechol O-methyl transferase and dopamine D_2_ receptor gene polymorphisms: evidence of positive heterosis and gene-gene interaction on working memory functioning. *Eur. J. Hum. Genet.* 16 1075–1082. 10.1038/ejhg.2008.57 18382477

[B42] Grady D. L. Chi H. C. Ding Y. C. Smith M. Wang E. Schuck S. (2003). High prevalence of rare dopamine receptor D4 alleles in children diagnosed with attention-deficit hyperactivity disorder. *Mol. Psychiatry* 8 536–545. 10.1038/sj.mp.4001350 12808433

[B43] Grossman S. R. Andersen K. G. Shlyakhter I. Tabrizi S. Winnicki S. Yen A. (2013). Identifying recent adaptations in large-scale genomic data. *Cell* 152 703–713. 10.1016/j.cell.2013.01.035 23415221 PMC3674781

[B44] Guo G. Roettger M. E. Shih J. C. (2007). Contributions of the DAT1 and DRD2 genes to serious and violent delinquency among adolescents and young adults. *Hum. Genet.* 121 125–136. 10.1007/s00439-006-0244-8 17120049

[B45] Hattori E. Nakajima M. Yamada K. Iwayama Y. Toyota T. Saitou N. (2009). Variable number of tandem repeat polymorphisms of DRD4: re-evaluation of selection hypothesis and analysis of association with schizophrenia. *Eur. J. Hum. Genet.* 17 793–801. 10.1038/ejhg.2008.247 19092778 PMC2947105

[B46] Hedrick P. W. (2006). Genetic polymorphism in heterogenous environments: the age of genomics. *Annu. Rev. Ecol. Evol. Syst.* 37 67–93. 10.1146/annurev.ecolsys.37.091305.110132

[B47] Hedrick P. W. (2012). What is the evidence for heterozygote advantage selection? *Trends Ecol. Evol.* 27 698–704. 10.1016/j.tree.2012.08.012 22975220

[B48] Homberg J. R. Lesch K. P. (2011). Looking on the bright side of serotonin transporter gene variation. *Biol. Psychiatry* 69 513–519. 10.1016/j.biopsych.2010.09.024 21047622

[B49] Iurescia S. Seripa D. Rinaldi M. (2015). Role of the 5-HTTLPR and SNP Promoter polymorphisms on serotonin transporter gene expression: a closer look at genetic architecture and in vitro functional studies of common and uncommon allelic variants. *Mol. Neurobiol.* 10.1007/s12035-015-9409-6 [Epub ahead of print]. 26464328

[B50] Jiang Y. Chew S. H. Ebstein R. P. (2013). The role of D4 receptor gene exon III polymorphisms in shaping human altruism and prosocial behavior. *Front. Hum. Neurosci.* 7:195 10.3389/fnhum.2013.00195 PMC3653059 23717276

[B51] Keller M. C. Miller G. (2006). Resolving the paradox of common, harmful, heritable mental disorders: which evolutionary genetic models work best? *Behav. Brain Sci.* 29 385–404. 10.1017/S0140525X06009095 17094843

[B52] Key F. M. Teixeira J. C. de Filippo C. Andrés A. M. (2014). Advantageous diversity maintained by balancing selection in humans. *Curr. Opin. Genet. Dev.* 29 45–51. 10.1016/j.gde.2014.08.001 25173959

[B53] Klopfstein S. Currat M. Excoffier L. (2006). The fate of mutations surfing on the wave of a range expansion. *Mol. Biol. Evol.* 23 482–490. 10.1093/molbev/msj057 16280540

[B54] Kreitman M. (2000). Methods to detect selection in populations with applications to the human. *Annu. Rev. Genomics Hum. Genet.* 1 539–559. 10.1146/annurev.genom.1.1.539 11701640

[B55] Lally J. MacCabe J. H. (2016). Personalized approaches to pharmacotherapy for schizophrenia. *Mol. Neurobiol.* 22 78–86.

[B56] Leffler E. M. Gao Z. Pfeifer S. Segurel L. Auton A. Venn O. (2013). Multiple instances of ancient balancing selection shared between humans and chimpanzees. *Science* 339 1578–1582. 10.1126/science.1234070 23413192 PMC3612375

[B57] Liu X. Ong R. T. Pillai E. N. Elzein A. M. Small K. S. Clark T. G. (2013). Detecting and characterizing genomic signatures of positive selection in global populations. *Am. J. Hum. Genet.* 92 866–881. 10.1016/j.ajhg.2013.04.021 23731540 PMC3675259

[B58] Livak K. J. Rogers J. Lichter J. B. (1995). Variability of dopamine D4 receptor (DRD4) gene sequence within and among nonhuman primate species. *Proc. Natl. Acad. Sci. U.S.A.* 92 427–431. 10.1073/pnas.92.2.427 7831304 PMC42753

[B59] Lopez Herraez D. Bauchet M. Tang K. Theunert C. Pugach I. Li J. (2009). Genetic variation and recent positive selection in worldwide human populations: evidence from nearly 1 million SNPs. *PLoS ONE* 4:e7888 10.1371/journal.pone.0007888 PMC2775638 19924308

[B60] MacDonald K. (1995). Evolution, the five-factor model, and levels of personality. *J. Pers.* 63 525–567. 10.1111/j.1467-6494.1995.tb00505.x

[B61] Matthews L. J. Butler P. M. (2011). Novelty-seeking DRD4 polymorphisms are associated with human migration distance out-of-Africa after controlling for neutral population gene structure. *Am. J. Phys. Anthropol.* 145 382–389. 10.1002/ajpa.21507 21469077

[B62] Maynard Smith J. (1982). *Evolution and the Theory of Games.* Cambridge: Cambridge University Press.

[B63] Mileva-Seitz V. R. Bakermans-Kranenburg M. J. van I. M. H. (2015). Genetic mechanisms of parenting. *Horm Behav.* 77 211–223. 26112881 10.1016/j.yhbeh.2015.06.003

[B64] Miller-Butterworth C. M. Kaplan J. R. Shaffer J. Devlin B. Manuck S. B. Ferrell R. E. (2008). Sequence variation in the primate dopamine transporter gene and its relationship to social dominance. *Mol. Biol. Evol.* 25 18–28. 10.1093/molbev/msm219 17934207

[B65] Mizuno H. Atwal G. Wang H. Levine A. J. Vazquez A. (2010). Fine-scale detection of population-specific linkage disequilibrium using haplotype entropy in the human genome. *BMC Genet.* 11:27 10.1186/1471-2156-11-27 PMC2873552 20416085

[B66] Montag C. Reuter M. (2014). Disentangling the molecular genetic basis of personality: from monoamines to neuropeptides. *Neurosci. Biobehav. Rev.* 43 228–239. 10.1016/j.neubiorev.2014.04.006 24769290

[B67] Mota N. R. Araujo-Jnr E. V. Paixao-Cortes V. R. Bortolini M. C. Bau C. H. (2012). Linking dopamine neurotransmission and neurogenesis: The evolutionary history of the NTAD (NCAM1-TTC12-ANKK1-DRD2) gene cluster. *Genet. Mol. Biol.* 35(4 Suppl.), 912–918. 10.1590/S1415-47572012000600004 23412349 PMC3571431

[B68] Murdoch J. D. Speed W. C. Pakstis A. J. Heffelfinger C. E. Kidd K. K. (2013). Worldwide population variation and haplotype analysis at the serotonin transporter gene SLC6A4 and implications for association studies. *Biol. Psychiatry* 74 879–889. 10.1016/j.biopsych.2013.02.006 23510579

[B69] Nachman M. W. Crowell S. L. (2000). Estimate of the mutation rate per nucleotide in humans. *Genetics* 156 297–304. 10978293 10.1093/genetics/156.1.297 PMC1461236

[B70] Naka I. Nishida N. Ohashi J. (2011). No evidence for strong recent positive selection favoring the 7 repeat allele of VNTR in the DRD4 gene. *PLoS ONE* 6:e24410 10.1371/journal.pone.0024410 PMC3164202 21909391

[B71] Nakamura M. Ueno S. Sano A. Tanabe H. (2000). The human serotonin transporter gene linked polymorphic region (5-HTTLP) shows ten novel allelic variants. *Mol. Psychiatry* 5 32–38. 10.1038/sj.mp.4000698 10673766

[B72] Nettle D. (2006). The evolution of personality variation in humans and other animals. *Am. Psychol.* 61 622–631. 10.1037/0003-066X.61.6.622 16953749

[B73] Nielsen R. Paul J. S. Albrechtsen A. Song Y. S. (2011). Genotype and SNP calling from next-generation sequencing data. *Nat. Rev. Genet.* 12 443–451. 10.1038/nrg2986 21587300 PMC3593722

[B74] Oleksyk T. K. Smith M. W. O’Brien S. J. (2010). Genome-wide scans for footprints of natural selection. *Philos. Trans. R. Soc. Lond. B Biol. Sci.* 365 185–205. 10.1098/rstb.2009.0219 20008396 PMC2842710

[B75] Penke L. Denissen J. J. A. Miller G. F. (2007). The evolutionary genetics of personality. *Eur. J. Pers.* 21 549–587. 10.1002/per.629

[B76] Pickrell J. K. Coop G. Novembre J. Kudaravalli S. Li J. Z. Absher D. (2009). Signals of recent positive selection in a worldwide sample of human populations. *Genome Res.* 19 826–837. 10.1101/gr.087577.108 19307593 PMC2675971

[B77] Qian W. Zhou H. Tang K. (2015). Recent coselection in human populations revealed by protein-protein interaction network. *Genome Biol. Evol.* 7 136–153. 10.1093/gbe/evu270 25532814 PMC4316623

[B78] Rafajlović M. Klassmann A. Eriksson A. Wiehe T. Mehlig B. (2014). Demography-adjusted tests of neutrality based on genome-wide SNP data. *Theor. Popul. Biol.* 95 1–12. 10.1016/j.tpb.2014.05.002 24911258

[B79] Sabol S. Z. Hu S. Hamer D. (1998). A functional polymorphism in the monoamine oxidase A gene promoter. *Hum. Genet.* 103 273–279. 10.1007/s004390050816 9799080

[B80] Scheinfeldt L. B. Tishkoff S. A. (2013). Recent human adaptation: genomic approaches, interpretation and insights. *Nat. Rev. Genet.* 14 692–702. 10.1038/nrg3604 24052086 PMC4882605

[B81] Slatkin M. Excoffier L. (2012). Serial founder effects during range expansion: a spatial analog of genetic drift. *Genetics* 191 171–181. 10.1534/genetics.112.139022 22367031 PMC3338258

[B82] Stein D. J. Newman T. K. Savitz J. Ramesar R. (2006). Warriors versus worriers: the role of COMT gene variants. *CNS Spectr.* 11 745–748. 17008817 10.1017/s1092852900014863

[B83] Sulik M. J. Eisenberg N. Spinrad T. L. Lemery-Chalfant K. Swann G. Silva K. M. (2015). Interactions among catechol-O-methyltransferase genotype, parenting, and sex predict children’s internalizing symptoms and inhibitory control: evidence for differential susceptibility. *Dev. Psychopathol.* 27 709–723. 10.1017/S0954579414000807 25159270 PMC4476935

[B84] Svardal H. Rueffler C. Hermisson J. (2015). A general condition for adaptive genetic polymorphism in temporally and spatially heterogeneous environments. *Theor. Popul. Biol.* 99 76–97. 10.1016/j.tpb.2014.11.002 25446960

[B85] Tajima F. (1989). Statistical method for testing the neutral mutation hypothesis by DNA polymorphism. *Genetics* 123 585–595. 2513255 10.1093/genetics/123.3.585 PMC1203831

[B86] Tishkoff S. A. Verrelli B. C. (2003). Patterns of human genetic diversity: implications for human evolutionary history and disease. *Annu. Rev. Genomics Hum. Genet.* 4 293–340. 10.1146/annurev.genom.4.070802.110226 14527305

[B87] van der Kooij M. A. Sandi C. (2015). The genetics of social hierarchies. *Curr. Opin. Behav. Sci.* 2 252–257.

[B88] Verweij K. J. Yang J. Lahti J. Veijola J. Hintsanen M. Pulkki-Raback L. (2012). Maintenance of genetic variation in human personality: testing evolutionary models by estimating heritability due to common causal variants and investigating the effect of distant inbreeding. *Evolution* 66 3238–3251. 10.1111/j.1558-5646.2012.01679.x 23025612 PMC3518920

[B89] Vitalis R. Gautier M. Dawson K. J. Beaumont M. A. (2014). Detecting and measuring selection from gene frequency data. *Genetics* 196 799–817. 10.1534/genetics.113.152991 24361938 PMC3948807

[B90] Vitti J. J. Grossman S. R. Sabeti P. C. (2013). Detecting natural selection in genomic data. *Annu. Rev. Genet.* 47 97–120. 10.1146/annurev-genet-111212-133526 24274750

[B91] Voight B. F. Kudaravalli S. Wen X. Pritchard J. K. (2006). A map of recent positive selection in the human genome. *PLoS Biol.* 4:e72 10.1371/journal.pbio.0040072 PMC1382018 16494531

[B92] Wall J. D. (2003). Estimating ancestral population sizes and divergence times. *Genetics* 163 395–404. 12586724 10.1093/genetics/163.1.395 PMC1462435

[B93] Wang E. Ding Y. C. Flodman P. Kidd J. R. Kidd K. K. Grady D. L. (2004). The genetic architecture of selection at the human dopamine receptor D4 (DRD4) gene locus. *Am. J. Hum. Genet.* 74 931–944. 10.1086/420854 15077199 PMC1181986

[B94] Wolf M. McNamara J. M. (2012). On the evolution of personalities via frequency-dependent selection. *Am. Nat.* 179 679–692. 10.1086/665656 22617258

[B95] Zhou H. Hu S. Matveev R. Yu Q. Li J. Khaitovich P. (2015). A chronological atlas of natural selection in the human genome during the past half-million years. *bioRxiv* 10.1101/018929

